# A review of the applications of generative adversarial networks to structural and functional MRI based diagnostic classification of brain disorders

**DOI:** 10.3389/fnins.2024.1333712

**Published:** 2024-04-15

**Authors:** Nguyen Huynh, Gopikrishna Deshpande

**Affiliations:** ^1^Auburn University Neuroimaging Center, Department of Electrical and Computer Engineering, Auburn University, Auburn, AL, United States; ^2^Department of Psychological Sciences, Auburn University, Auburn, AL, United States; ^3^Alabama Advanced Imaging Consortium, Birmingham, AL, United States; ^4^Center for Neuroscience, Auburn University, Auburn, AL, United States; ^5^Department of Psychiatry, National Institute of Mental Health and Neurosciences, Bangalore, India; ^6^Department of Heritage Science and Technology, Indian Institute of Technology, Hyderabad, India

**Keywords:** generative adversarial network (GAN), classification, fMRI, brain connectivity, deep learning

## Abstract

Structural and functional MRI (magnetic resonance imaging) based diagnostic classification using machine learning has long held promise, but there are many roadblocks to achieving their potential. While traditional machine learning models suffered from their inability to capture the complex non-linear mapping, deep learning models tend to overfit the model. This is because there is data scarcity and imbalanced classes in neuroimaging; it is expensive to acquire data from human subjects and even more so in clinical populations. Due to their ability to augment data by learning underlying distributions, generative adversarial networks (GAN) provide a potential solution to this problem. Here, we provide a methodological primer on GANs and review the applications of GANs to classification of mental health disorders from neuroimaging data such as functional MRI and showcase the progress made thus far. We also highlight gaps in methodology as well as interpretability that are yet to be addressed. This provides directions about how the field can move forward. We suggest that since there are a range of methodological choices available to users, it is critical for users to interact with method developers so that the latter can tailor their development according to the users' needs. The field can be enriched by such synthesis between method developers and users in neuroimaging.

## 1 Introduction

Structural and functional magnetic resonance imaging have held promise as a potential biomarker for diagnosing patients with neurological and neuropsychiatric conditions. Functional magnetic resonance imaging (fMRI), a technique that detects changes in blood flow associated with increased neural activity, has been found informative in identifying functional impairments in brain disorders. Resting-state fMRI (rs-fMRI), which examines the spontaneous fluctuations of blood-oxygen levels in the absence of neuronal stimulation, has been increasingly utilized as a biomarker for various brain disorders. This technique explores the intrinsic functional characteristics of the brain network, revealing alterations in functional connectivity (FC) (can be expressed in terms of the statistical relationship between two time series from different brain regions) in patients with brain disorders compared to healthy individuals. For instance, research on autism spectrum disorder revealed a reduction in FC between the posterior superior temporal sulcus and amygdala, linked to voice perception and language development, as discovered by Alaerts et al. (2015). Additionally, Gotts et al. (2012) showed decreases in limbic-related brain regions associated with social behavior, language and communication. FC abnormalities also have been reported in patients with Alzheimer's disease (AD) when compared to healthy controls, particularly within the default mode network (DMN), a network that is involved in memory tasks among other functions. These disruptions occur between the precuneus and posterior cingulate cortex with the anterior cingulate cortex and the medial prefrontal cortex, as indicated by Brier et al. (2012) and Griffanti et al. (2015). Machine learning (ML) or deep learning algorithms have been extensively used to improve diagnostic results, leveraging the FC abnormalities in patients as informative features. These algorithms have been proven to significantly contribute to more accurate and early detection of brain disorders (Deshpande et al., 2015; Rabeh et al., 2016; Zou et al., 2017; Qureshi et al., 2019; Lanka et al., 2020; Ma et al., 2021; Yan et al., 2021), providing a rapid and automated tool for future diagnostic applications.

Beside fMRI, structural or anatomical MRI can also be useful in ML applications by offering morphometric information about the brain, such as volumes of white matter (WM) and gray matter (GM), cortical thickness, etc. Measuring hippocampal volume, a metric derived from structural MRI, has been shown to discriminate not only between AD patients and healthy subjects, but also among individuals with other dementia-related disorders (Schuff et al., 2009; Vijayakumar and Vijayakumar, 2013). Diffusion tensor imaging (DTI) is one of the modality that can reveal structural information about brain connectivity by measuring the direction and magnitude of water diffusion. DTI has the capability to define structural connectivity (SC) based on the fibers that link each pair of brain regions. Research has demonstrated that analyzing connectivity patterns through DTI-based SC can effectively distinguish individuals with brain disorders. This approach offers valuable insights into the irregularities with neural pathways, contributing to the identification and understanding of various neurological conditions (Tae et al., 2018; Billeci et al., 2020). Many studies have illustrated that enhancing diagnostic accuracy is achievable by incorporating multimodal information from both functional and structural connectivity data (Libero et al., 2015; Pan and Wang, 2022; Cao et al., 2023). However, the practical application of ML is still impeded by challenges such as high-dimensional spaces and imbalanced datasets in real-world scenarios. In addressing these obstacles, the implementation of generative adversarial network has shown promise, offering a potential solution to mitigate these issues and enhance the effectiveness of ML approaches.

Generative adversarial network (GAN) was proposed by Goodfellow et al. (2014). The concept is inspired by the zero-sum game in game theory (where one agent's gain is another agent's loss). GAN is a generative model that learns to produce synthetic images from random noise *z* derived from the prior distribution *p*(*z*), which is commonly Gaussian or uniform distribution. With the impressive performance shown in image generation, its unique and inspiring adversarial characteristic to discover data distributions is also exploited in clinical applications wherein GAN is being used for classification, detection, segmentation, registration, de-noising, reconstruction and synthesis. Here, we provide a brief overview of the vanilla GAN (the original GAN) and its extensions that have been developed and commonly used in clinical applications to brain disorders.

Data scarcity and imbalanced data (the number of healthy individuals often exceeds the number of unhealthy ones) are common challenging issues in classification task. Traditional data augmentation methods such as flipping, rotation, cropping, scaling and translation generate data sharing a similar distribution with the original ones, leading to the performance of the model does not improve. Since the topology of brain networks plays a pivotal role in characterizing information flow and communication between different brain regions, these methods, while effective for regular image data, can inadvertently distort the connectivity patterns encoded in FC brain network data. While certain data augmentation techniques, such as Synthetic Minority Over-Sampling (SMOTE) (Eslami and Saeed, 2019; Eslami et al., 2019) or Adaptive synthetic sampling (ADASYN) (Koh et al., 2020; Wang et al., 2020) has been proposed tackle the challenge of data imbalance, they often rely on linear interpolation for data sampling. This may introduce artifacts as well as data redundancies. Consequently, these methods might not be optimally effective for robust data augmentation. GAN can be used as an alternative data augmentation technique and it have been proved to improve the performance of the model. Zhou et al. (2021) proposed a GAN model that can generates 3 T imaging data from 1.5 T data collected from Alzheimer's Disease Neuroimaging Initiative (ADNI) dataset and they used both the real 3 T and synthetic 3 T data to classify healthy subjects with Alzheimer's patients. However, to apply 2D convolution of CNN in more appropriate way, Kang et al. (2021) divided the 3D images into various 2D slices and applied ensemble method to produce the best results.

However, those methods are still applied on 3D imaging data, which cost a large amount of computational resources. To solve this problem, a GAN framework (Yan et al., 2021) was proposed with the generator that can take a random noise input and produce synthesis functional connectivity (FC). The model used the BrainNetCNN (Kawahara et al., 2017) as discriminator to extract meaningful features and it can perform two tasks simultaneously: testing the authenticity of the output data and classifying which label the data belong to. Other approaches also generated FC constructed from independent component analysis (Zhao et al., 2020) or combined variational autoencoder (VAE) with GAN (Geng et al., 2020) to give more control to the latent vectors.

There are also a number of obstacles occurring when we train a GAN model. Mode collapse is one of the most difficult problem in training GAN model. This phenomenon occurs when the generator continually produces a similar image and the discriminator fails to distinguish the real and fake samples generated by the generator. To solve this problem, we can incorporate the subject's attribute data to the latent noise input, as we have done in Yan et al. (2021). By doing this, we add more information to the prior distribution, hence the improvement in the diversity of the generated samples. The objection function has been proven as optimizing the Jensen-Sharon (JS) divergence, which is a difficult point to achieve when training a GAN model. We can mitigate this problem by using Wasserstein loss function, which has proved to increase the stability of GAN training (Arjovsky et al., 2017).

Heterogeneous domain adaptation task is a problem that leverages the labeled data from the source domain to learn the data from other domains (target domains). Deep neural networks excel at learning from the data that they were trained on, but perform poorly at generalizing learned knowledge to new datasets. Cycle-consistent GAN (CycleGAN) (Zhu et al., 2017) can be used to learn the mapping between the two domains and minimize the distance between the source and target feature distributions. Therefore, it can be a potential solution to transfer knowledge from different open-source datasets, which contains data with different acquisition protocols of the scanner (Wollmann et al., 2018) applied CycleGAN to perform classification task and experimental results shown that domain adaptation method produces better accuracy than state-of-the-art data augmentation technique. This domain adaptation method is still at its early stage and further research is needed to explore CycleGAN for the classification of brain disorders using data from different domains.

From the brief introduction above, it is clear that GANs hold immense potential for application to neuroimaging-based diagnosis of brain disorders wherein it can be deployed to solve some problems unique to neuroimaging as well. Therefore, we provide a review of the applications of GAN to neuroimaging-based diagnosis and identify potential problems and future directions. Before we do so, a brief methodological primer is provided on GANs so that the readers can appreciate the range of methodological choices available to end users that may be more appropriate for their given applications. Next, we delve into an extensive exploration of the applications of these models, particularly in the context of brain disorder diagnostics using functional/structural MRI data. The primary aim is to elucidate the observable impact of GANs as a data augmentation method in this critical diagnostic domain.

## 2 Methodological primer

### 2.1 Vanilla GAN

GAN consists of two models that are trained simultaneously in an unsupervised way. The two models are called the discriminator (D) and the generator (G). The goal of D is to test the authenticity of the data (real or fake) while G's objective is to confuse D as much as possible. We can view that each time G make a poor product, D will send a signal to inform G to improve the product. When G improves its product's quality, D will also try to better penetrate it an this in turn causes G to improve its product to an even higher level. Therefore, we can see this process as a min-max operation.

Mathematically, assuming that D and G are neural networks parameterized by θ_*d*_, θ_*g*_, G can be seen as a non-linear mapping function that generates $\hat{x}=\text{G}\left(z;\theta_{g}\right)$ from random noise *z* drawn from a prior distribution *p*_*z*_ (*z*~*p*_*z*_(*z*)) and $\hat{x}$ is supposed to follow the distribution $p_{\theta }\left(\hat{x}|z\right)$. On the other hand, the output of D is just a label that indicates whether the input is a real or fake sample *y* = *D*(*x*; θ_*D*_) or in other words, D is a binary classifier. Given real data following the distribution *p*_real_(*x*), the main purpose of training the GAN model is to form the distribution of the generated sample to approximate the distribution of the real data: $p_{\theta }\left(\hat{x}|z\right)\approx p_{\text{real}}$. In other words, D can no longer distinguish the fake product generated by G. The loss functions to train those two models can be calculated as Equation 1 and Equation 2:


(1) $$\begin{array}{l} \begin{array}{l} L_{D}=\underset{D}{\max}{𝔼}_{x\sim p_{\text{real}}\left(x\right)}\left[\log D\left(x\right)\right]\\ +{𝔼}_{\hat{x}\sim p_{\theta }\left(\hat{x}|z\right)}\left[\log\left(1-D\left(\hat{x}\right)\right)\right] \end{array} \end{array}$$

(2) $$\begin{array}{l} L_{G}=\underset{G}{\min}{𝔼}_{\hat{x}\sim p_{\theta }\left(\hat{x}|z\right)}\left[\log\left(1-D\left(\hat{x}\right)\right)\right] \end{array}$$

The training procedure has been proven to be equivalent to minimizing the Jensen-Shannon divergence between the distribution of real and synthetic data. The models also employ back propagation to update their parameters. When the discriminator is undergo training, the parameters of G are fixed. The discriminator D receives both the real data x (positive sample) and the generator's fake data $\hat{x}$ (negative sample) as inputs and the error used for back propagation is calculated by the output of D and the sampled data. Similarly, when training G, the parameters of D are fixed. The sample data generated by G is labeled as fake and fed into the discriminator. The output of the discriminator *D*(*G*(*z*; θ_*G*_)) and the labeled data from G are used to calculate the error used for the back propagation algorithm. The parameters of D and G are continuously updated by those steps until we reach the equilibrium.

### 2.2 InfoGAN

**Figure 1:**
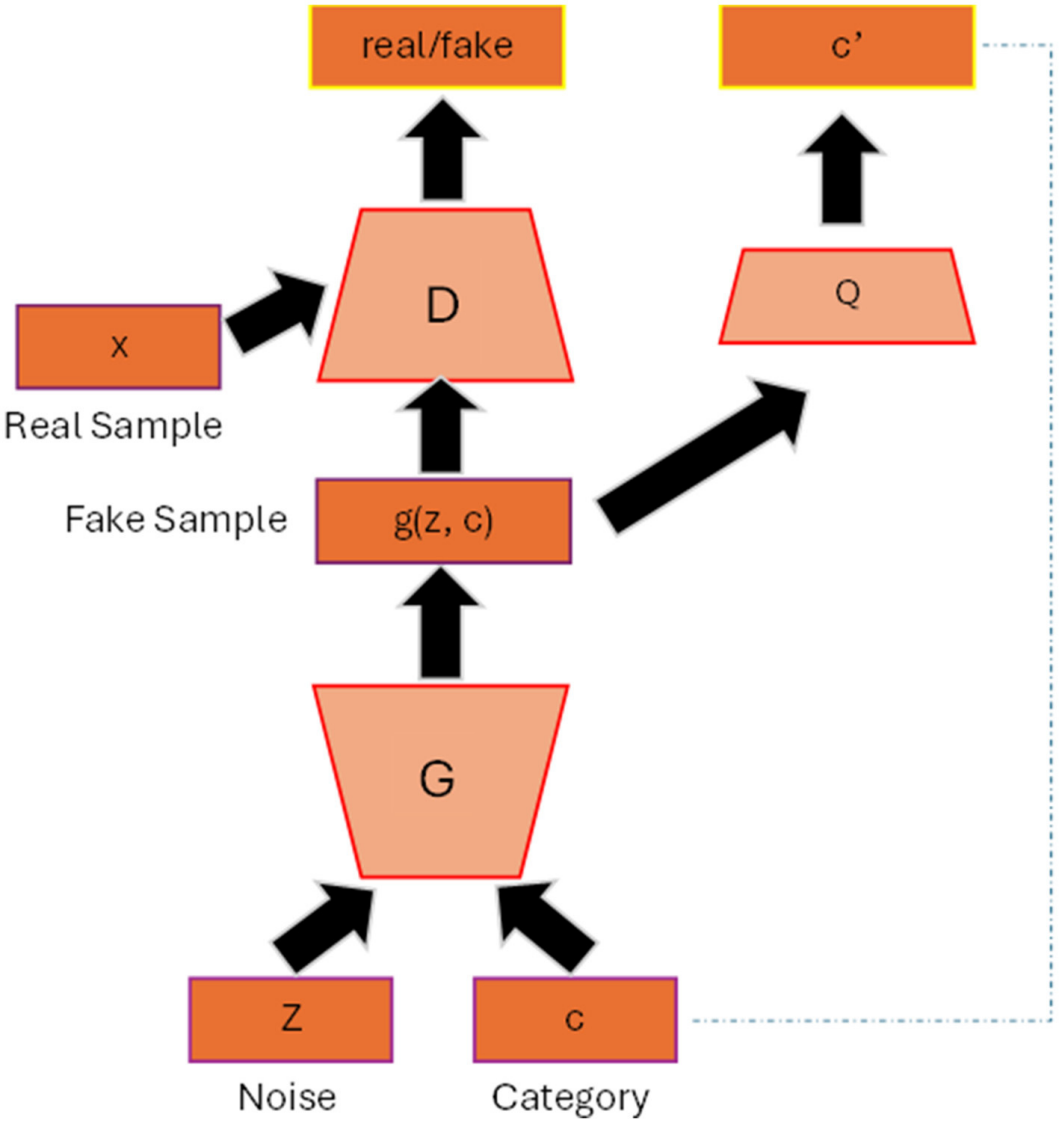
InfoGAN architecture. The data attribute c is added to the input of the generator. The Q classifier uses the generated data from G as input and produces the information distribution c′ that resembles c.

In the original GAN, the generated images from the generator are totally random and there is no control regarding the properties of the images. InfoGAN (Chen et al., 2016) helps the generator to have a better control of the generated output by involving its mutual information (typically data attributes of the images) to the latent vector. For example, to have a better quality of face images we also need other factors such as the shape of the eyes, hair style and hair color, etc. Figure 1 shows the general architecture of infoGAN. In infoGAN, the problem is to maximize the data distribution of generated output and the latent attribute vector. Therefore, the loss function of the standard GAN will includes the information term as regularization which can be seen in Equation 3:


(3) $$\begin{array}{l} \underset{G}{\min}\underset{D}{\max}L_{I}\left(D,G\right)=L\left(D,G\right)-\lambda I\left(c;G\left(z,c\right)\right) \end{array}$$

where λ*I*(*c*; *G*(*z, c*)) is the mutual information term.

### 2.3 Conditional GAN

**Figure 2:**
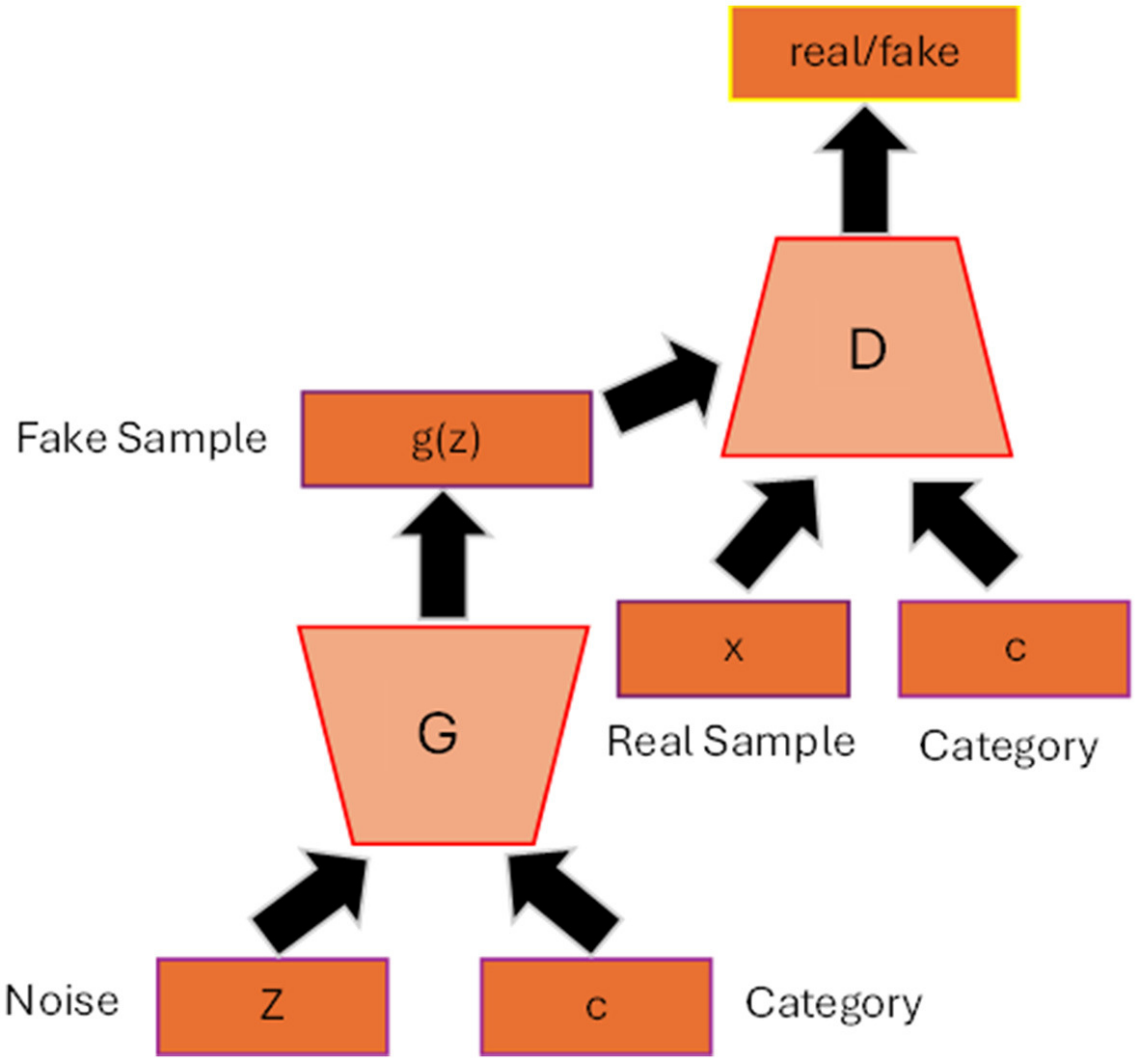
Conditional GAN architecture. The label c is added as input to the generator and discriminator so that the generator can generate synthetic data belonging to that specific label.

Figure 2 shows the general architecture of conditional GAN (cGAN) (Mirza and Osindero, 2014). By adding the auxiliary information **c** to the generator and discriminator such as class label, the model can generate data that belongs to that specific label. The conditioned information not only guides the generator to produce high quality synthesis image but also helps to improve the stability of the training process. The loss function will then include the conditioned **c** as depicted in Equation 4 and Equation 5:


(4) $$\begin{array}{l} \begin{array}{l} L_{D}=\underset{D}{\max}{𝔼}_{x\sim p_{\text{real}}\left(x\right)}\left[\log D\left(x|\text{c}\right)\right]\\ +{𝔼}_{\hat{x}\sim p_{\theta }\left(\hat{x}|z\right)}\left[\log\left(1-D\left(G\left(z|\text{c}\right)\right)\right)\right] \end{array} \end{array}$$

(5) $$\begin{array}{l} L_{G}=\underset{G}{\min}{𝔼}_{\hat{x}\sim p_{\theta }\left(\hat{x}|z\right)}\left[\log\left(1-D\left(G\left(z|\text{c}\right)\right)\right)\right] \end{array}$$

### 2.4 AC-GAN

The auxiliary classifier GAN (AC-GAN) (Odena et al., 2017) is an extension of the cGAN where the discriminator is slightly modified so that it can also provide the prediction of the class label along with the authenticity of the input. In particular, similar to cGAN, the generator will also receive the class label combined with the latent vector as input, while the discriminator is provided with only the images, instead of both the images and class label in cGAN. The discriminator will add one more head that uses softmax activation function to provide the probability for each class label, enabling GAN to performance classification task.

### 2.5 Wasserstein GAN

Wasserstein GAN (WGAN) (Arjovsky et al., 2017) was proposed to deal with the common issues that often occur when training the GAN model, such as mode collapse or JS divergence. The paper introduces the new distance metric—Earth Moving distance or Wasserstein distance that can be formulated as in Equation 6:


(6) $$\begin{array}{l} W\left({\mathbb{P}}_{r},{\mathbb{P}}_{\theta }\right)=\underset{∥f{∥}_{L}\leq 1}{\sup}{𝔼}_{x\sim {\mathbb{P}}_{r}}\left[f\left(x\right)\right]-{𝔼}_{x\sim {\mathbb{P}}_{\theta }}\left[f\left(x\right)\right] \end{array}$$

where *f* is a 1-Lipschitz function. To solve this equation, we can model *f* as a neural network and learn the parameters from it. The solution can be shortly summarized as in Equation 7:


(7) $$\begin{array}{l} \underset{w\in W}{\max}{𝔼}_{x\sim {\mathbb{P}}_{r}}\left[f_{w}\left(x\right)\right]-{𝔼}_{z\sim p_{z}}\left[f_{w}\left(g_{\theta }\left(z\right)\right)\right] \end{array}$$

Initially, the model used weight clipping technique to satisfy the Lipschitz constraint that may lead to vanishing gradient problem. The paper later introduced a gradient penalty technique to enforce Lipschitz constraint to improve the stability of the training process.

### 2.6 CycleGAN

CycleGAN (Zhu et al., 2017) is one of the most commonly used models in the generation of medical images due to its capability to perform cross-modality transition, such as synthesizing brain CT images from MRI images. CycleGAN consists of two generators and two discriminators where each generator will receive a set of data from other modality and the data are not necessary to pair with each other. Let us assume that we have data samples *x*∈*X* and *y*∈*Y* where *X, Y* are training data for which we want to make a transition and G, F are two generators or two translators that make a mapping: ŷ = *G*(*x*) and $\hat{x}=F\left(y\right)$. If we use the original GAN's loss function to update the model, the generator may be able to generate data in each domain, but it is not sufficient to generate translations of the input data. CycleGAN introduces an additional concept called cycle consistency which states that the transitions between the two translators are bijections, meaning that *F*(*G*(*x*)) = *x* and *G*(*F*(*y*)) = *y*. The cycle consistent loss is added to the original loss function as the regularization term as shown in Equation 8:


(8) $$\begin{array}{l} \begin{array}{l} L_{\text{cyc}}\left(G,F\right)={𝔼}_{x\sim p_{\text{data}}\left(x\right)}\left[∥F\left(G\left(x\right)\right)x{∥}_{1}\right]\\ +{𝔼}_{y\sim p_{\text{data}}\left(y\right)}\left[∥G\left(F\left(y\right)\right)-y{∥}_{1}\right] \end{array} \end{array}$$

**Figure 3:**
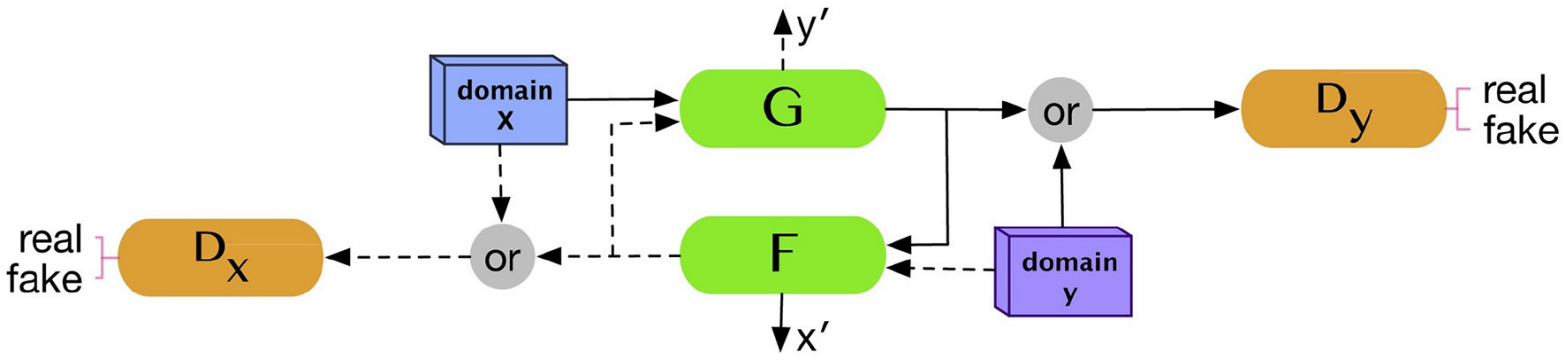
CycleGAN architecture. Two generators and two discriminators are trained in the CycleGAN model. Each generator receives a set of data from the other domain to produce synthetic data from its domain. Reprinted from: Kazeminia et al. (2020). Copyright (2020) with permission from Elsevier.

Figure 3 illustrated the overall architecture of CycleGAN.

## 3 Similarity metrics

The synthetic data must undergo validation against real data to assess the model's effectiveness. Numerous similarity metrics have been developed to evaluate the features of real and synthesized brain networks. This section provides a brief overview of these techniques that have been used in previous literature.

**Kullback-Leibler Divergence (KL Divergence)** measures the difference between two probability distributions p(x) and q(x) [where p(x) and q(x) are the probability of the data x ∈ X occurring in the real and fake data distribution, respectively]. The formula for KL divergence can be expressed as Equation 9:


(9) $$\begin{array}{l} \text{KL}\left(p\left(x\right)||q\left(x\right)\right)=\underset{x\in X}{\sum }\ln\;p\left(x\right)\frac{p\left(x\right)}{q\left(x\right)} \end{array}$$

**Maximum Mean Discrepancy (MMD)** quantifies distribution differences by measuring the distances between distributions through their respective mean feature embeddings, which can be expressed as Equation 10:


(10) $$\begin{array}{l} \text{MMD}\left(P,Q\right)=||{𝔼}_{X\sim P}\left[\phi \left(x\right)\right]-{𝔼}_{Y\sim Q}\left[\phi \left(Y\right)\right]|{|}^{2} \end{array}$$

where X and Y are the sample from real distribution P and fake distribution Q, respectively. ϕ is a kernel function that maps X and Y into a higher-dimensional space.

**Graph-based global metrics:** Transitivity, global efficiency, modularity, density, betweenness centrality, and assortativity (Beauchene et al., 2018) are prevalent metrics employed for quantifying meaningful graph attributes and therefore serving as tools to assess the quality of generated graphs.

## 4 Applications of GAN for brain disorder classification

One of the most challenging problems in training a deep learning model for brain disorder classification is the small amount of data often causing the model to be overfitted. One possible solution to this problem is using GAN for data augmentation due to its capability to synthesize high-quality images that resemble real MRI data. Many researches have shown that using generated images from GAN can assist the training process and improve the classification performance (Shin et al., 2018). In this work, we will focus on the discussion of the GAN models that utilize synthetic MRI images to assist the classification problem.

Zhou et al. (2021) used both GAN and fully convolutional network (FCN) to generate 3 T MRI images from 1.5 T images in the ADNI dataset to distinguish the brain images of AD patients from those of the healthy group. The goal of the generator is to use 1.5 T data as the input to generate 3 T images (called 3 T^*^) images with better resolution to improve the classification performance. This method is similar to residual learning, where the model tries to figure out the missing features or the differences between two types of images and the model will add those features to transform 1.5–3T images. The main advantages of this method is that by learning only the residual, we could save a large amount of computational resources and the model could learn the important features more easily.

Training GAN model for the 3D images requires a large amount of data to avoid over-fitting problem. Furthermore, it may not be appropriate for the 2D CNN to be trained on 3D data. Therefore, Kang et al. (2021) suggested to train on multiple 2D slices selected from the coronal axis and then proposed a major voting scheme to obtain best accuracy results. The model was first trained on all the slices with deep convolutional GAN (DCGAN) method (Radford et al., 2015). Then they used transfer learning that froze the few first layers' trainable weights and fine-tuned the weights of the remaining layers on three models: the pretrained DCGAN, VGG16 (Simonyan and Zisserman, 2014) and ResNet50 (He et al., 2016) to select the models that produce the best results. VGG16 and ResNet50 were pretrained on the ImageNet dataset. The results have shown that ensemble learning with three classifiers is more stable and has the highest performance than when training with only one classifier.

Most of the works focus on generating 3-D brain images which demands huge computational resources as well as lacks interpretability. A GAN model that can produce synthetic functional connectivity can be more effective for tackling both of these problems. Geng et al. (2020) proposed a brain functional connectivity generative adversarial network (FC-GAN) that combines variational autoencoder (VAE) and adversarial strategy to generate FC data from the original dataset. The model consists of three components: the encoder took the real data as input to generate the mean and variance of the latent code that follows the normal distribution and use it as a noise vector; the decoder took the noise vector to generate fake data, which is equivalent to the generator component in GAN; and finally the discriminator was designed based on WGAN loss that used Wesserstein distance to calculate the difference between fake and real data. Those three components were jointly connected and trained separately. The whole model was trained in two steps: the first step was trained according to traditional GAN procedure to receive augmented FC data. Then the augmented data was combined with the experimental data and they were fed into the deep neural network (DNN) containing multiple fully connected layers to output the class for each subject. The experimental results on ASD (256 HC and 198 patients with AD), ADHD (272 HC and 215 patients with ADHD), and ASD-ABIDE datasets (829 HC and 660 patients with AD) show the improvement in accuracy when training with DNN alone (87.16, 87.27,and 70.22%, respectively, compared to 85.35, 85.06, and 67.22%, respectively).

**Figure 4:**
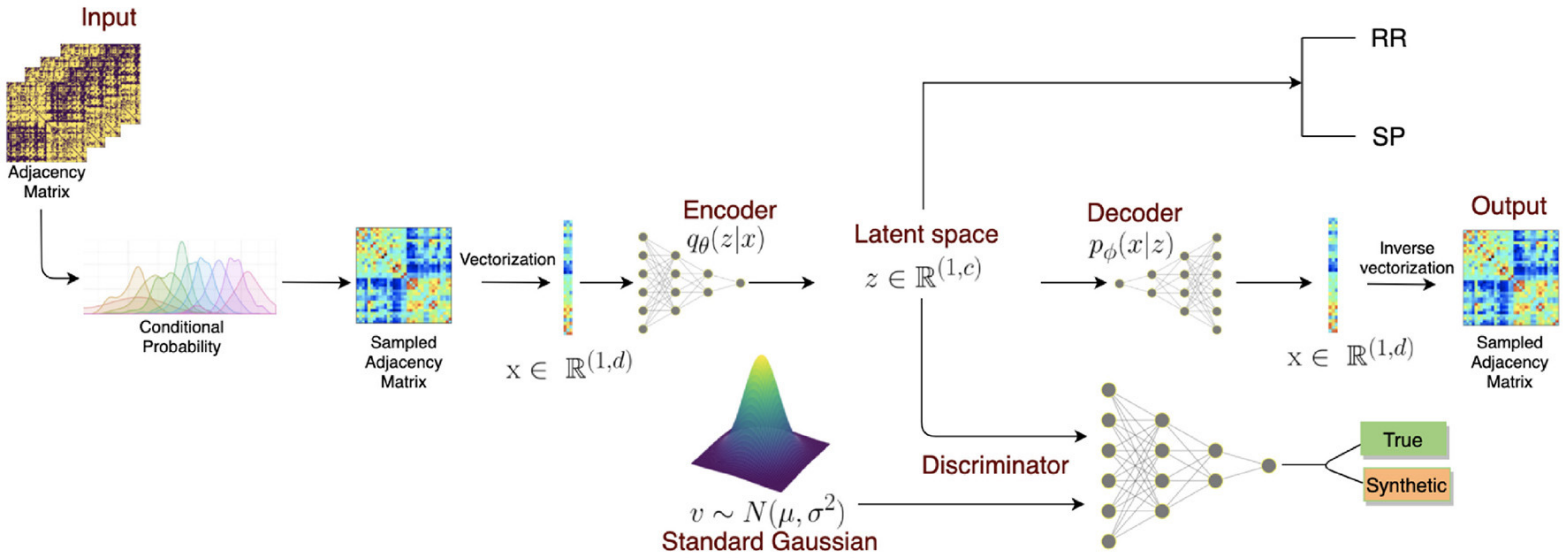
VAE + GAN end-to-end architecture. The model consists of three parts: (1) an encoder transforms the sampled adjacency matrix inputs to a latent lower dimensional representation. (2) The decoder aims to reconstruct the original input from the latent representation *z*. (3) The discriminator takes both the latent representation and random noise vector as inputs and tries to discriminate these distributions. Once the model is trained, the model can generate synthetic graphs from a standard Gaussian distribution which then can be combined with experimental data for classification. Reprinted from: Barile et al. (2021). Copyright (2021) with permission from Elsevier.

GAN with autoencoder was also applied in generating brain connectivity for the classification of multiple sclerosis (MS) (Barile et al., 2021) (Figure 4). However, the output of the encoder will try to resemble the data drawn from the normal distribution instead of the image data. The model demonstrated an improvement of accuracy when training with the generated connectomes than with the original ones, meaning that the model could generate meaningful connections that could help to identify the disease. The model was tested on Multiple Sclerosis (MS) dataset, which included 29 relapsing-remitting and 19 secondary-progressive MS patients, achieving the highest F1-score of 81% compared to only 65.65% without using a data augmentation method. The model also outperformed other data augmentation techniques by margins exceeding over 10% (72.32% for SMOTE and 64.84% for the Random Over Sampling (ROS) method).

Another FC-based method that also applied a GAN model for the classification of major depressive disorder (MDD) patients from HC was proposed by Zhao et al. (2020). The model implemented a feature mapping technique in GAN, which is a specific method that can increase the efficiency of the training process of the GAN model (Salimans et al., 2016). The feature mapping method changes the way of training the generator where the discriminator will guide the generator to match the features of the intermediate layer of the discriminator. The new objective function for the generator can be defined as Equation 11:


(11) $$\begin{array}{l} ∥E_{x\sim p_{data}}f\left(x\right)-E_{Z\sim p_{Z}\left(z\right)}f\left(G\left(z\right)\right){∥}_{2}^{2} \end{array}$$

If the model is trained by this method, the generator can produce data that match the statistic of the real data, avoiding model collapse during training. The author also proposed the leave-one-FNC-out method to determine which components or brain regions are important for predicting the disease. In particular, by observing the drop in the model's performance when removing a component, the potential biomarkers can be identified. The model underwent testing on two datasets: the MDD dataset, consisting of 269 MDD and 286 healthy controls (HC) and the Schizophrenia dataset, comprising 558 schizoprenia patients and 542 HC. The model achieved an accuracy of 70.1% for the MDD dataset and 80.7% for the Schizophrenia dataset, respectively. These results were at least 6 and 3–6% higher, compared to the performance of 6 other traditional ML approaches.

Furthermore, for the purpose of enhancing the quality of generated data, Cao et al. (2023) introduced a multiloop algorithm. This algorithm enables the assessment and ranking of the sample distribution in each iteration, allowing for the selection of solely high quality samples for training. The paper additionally suggests training the data using PatchGAN (Isola et al., 2017), a technique extensively employed in computer vision to extract features from small patches. This method offers the advantage of enabling the model to focus more on local details, which in turn enhances the similarly between real and synthetic data. The paper continued the training of the model by employing Wasserstein loss with gradient penalty method, which helped prevent the discriminator and generator from diverging. The effectiveness of the proposed model is evident in its evaluation on the AD dataset, which comprises 42 HC and 42 patients with AD. The proposed model displayed a higher accuracy (83.8%) compared to the models that were either trained without augmented data and loops (81.4%), or solely employed conditional GANs (81.6%).

Another model that was proposed to generate brain network connectivity is called as BrainNetGAN (Li C. et al., 2021) that includes three parts: a generator, a discriminator and a classifier. The generator consists of multiple fully connected layers and a reshape layer that converts the combined input of the random noise vector and one-hot label vector to brain network matrix. Both the discriminator and the classifier adopted the architecture of BrainCNN model (Kawahara et al., 2017) that design layers with specialized kernels, namely edge-to-edge layer, edge-to-node layer and node-to-graph layer. The discriminator is responsible for classifying real and fake matrices with the loss function adopted from WGAN to increase the stability during training, while the classifier used the binary cross entropy loss to classify whether the subject is healthy or not. The model was evaluated for dementia classification with AD dataset including 110 patients with AD and 110 HC. When trained using a combination of real and synthetic data, it attained an accuracy of 85.2%, outperforming the baseline model's accuracy of 81.9%. Furthermore, it demonstrated superiority over data augmentation methods, including SMOTE (82%) and ADASYN (77.8%). In terms of the quality of generated data, BrainNetGAN also achieved the closest similarity between real and fake data, with an average KL divergence score of 0.26 and a MMD score of 0.017. In comparison, SMOTE and ADASYN scored 0.51 and 0.53, respectively for KL divergence, and 0.053 and 0.06 for MMD.

The models mentioned above utilized inner production between paired node features to construct graph brain networks, potentially neglecting the intricate topological characteristics of brain networks. Therefore, by taking into consideration information from neighboring nodes, a Hemisphere-separated Cross Connectome Aggregating Learning (HCAL) model was designed (Zuo et al., 2023) to produce high-quality graphs. In particular, a rough connectivity matrix **A** was first divided into four sub-matrices: left hemisphere, right hemisphere and two interhemisphere. These sub-matrices were subsequently processed through a specially designed Cross-connectome Aggregating (CCA) module, which can incorporate the impacts of nodes within each network. Afterward, the four new matrices are combined to form the connectivity matrix **A_2_**, which is then subjected to a final CCA process aimed at extracting global topological features. The effectiveness of the proposed model was assessed on ADNI dataset, consisting of 135 HC and 135 patients with early mild cognitive impairment (EMCI), using the distance distribution metric to quantify disparities between raw and generated model, achieving the smallest value of 0.24 in comparison with 1.1 from BrainNetGAN and 0.4 from the adversarial regularized graph autoencoder (ARAE) method. The results highlights its efficacy in generating data that preserves similar characteristics with diverse structural features, an aspect important for improving diagnostic accuracy. The generated data was combined with the raw data for AD classification, achieving an accuracy of 84.48%. This outperformed the model's performance without augmented data (71.18%) and even surpassed other augmentation algorithms such as SMOTE (75.62%) and BrainNetGAN (81.29%).

**Figure 5:**
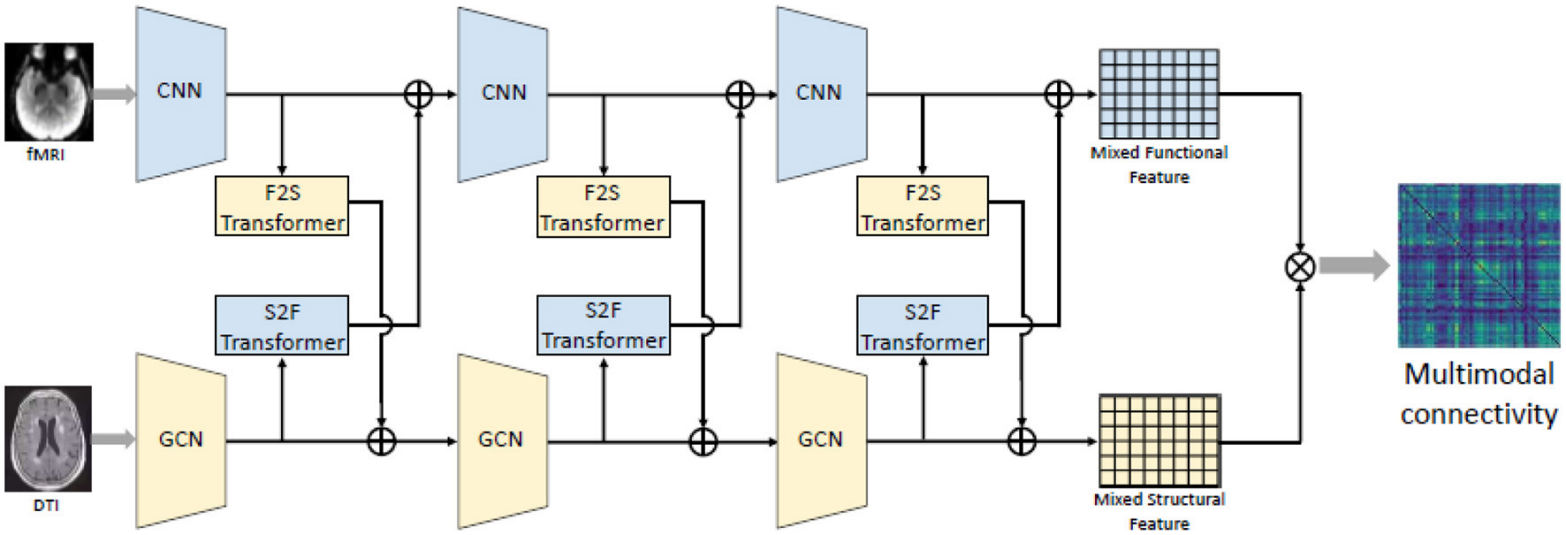
Illustration of the fusion scheme between FC and SC using bi-attention mechanism proposed by Pan and Wang (2022). The resting-state fMRI' feature sequences were extracted by the CNN model while the structural connective features were first extracted by the GCN model. Then multiple transformers was used to map complementary information between functional and structural matrix, resulting a mixed functional-structural output. Source: Pan and Wang (2022). Reproduced with permission.

Numerous studies have identified spatial overlaps between structural connectivity (SC) and functional connectivity in the brains of patients with brain diseases, revealing pathological features (Schultz et al., 2012; Rudie et al., 2013; Hua et al., 2020). Hence, a fusion scheme among modalities can yield enhanced feature representations for machine learning models. Pan and Wang (2022) suggested a bi-attention mechanism originally used in Transformer model for natural language processing (NLP) to effectively extract structural-functional information. The described approach can be summarized in Figure 5. Similar to the self-attention approach used in Transformer model, a matrix input $X\in R^{n\times d_{x}}$ (n is the number of ROI and *d*_*x*_ is the number of features for each ROI) is first transformed to a query, key and value *Q, K, V* by linear projections as indicated in Equation 12:


(12) $$\begin{array}{l} Q=XW^{q}\;K=XW^{k}\;V=XW^{v} \end{array}$$

where $W^{q}\in {\text{R}}^{d_{x}\times d_{q}},W^{k}\in {\text{R}}^{d_{x}\times d_{k}}$, (*d*_*q*_ = *d*_*k*_), and $W^{v}\in {\text{R}}^{d_{x}\times d_{v}}$ are the weight matrices. The attention output can then be calculated as Equation 13:


(13) $$\begin{array}{l} \text{Attention}\left(Q,K,V\right)=\text{softmax}\left(\frac{QK^{T}}{\sqrt{d_{k}}}\right)V \end{array}$$

then a fully connected layer was used to transform the output the same space as the target matrix $Y\in {\text{R}}^{n\times d_{y}}$:


(14) $$\begin{array}{l} X^{out}=\text{FullyConnected}\;\left(\text{Attention}\left(Q,K,V\right)\right)+\lambda Y \end{array}$$

where λ is a hyperparameter between 0 and 1. *X* and *Y* can be either functional or structural information Equation 14 depicts this transform. Then after multiple transformers, the final mixed output of FC and SC can be achieved as we can see in Figure 5.

The end-to-end framework was trained by incorporating the pairwise reconstruction loss alongside the adversarial loss and auxiliary classification loss to increase stability during training. The proposed GAN model, featuring bi-attention mechanism, when tested on the ADNI dataset, including 63 patients with AD, 41 patients with late mild cognitive impairment (LMCI), 80 EMCI and 84 HC, demonstrated superior performance compared to the the SVM model, the CNN model with transfer learning and the GCN model in terms of accuracy, sensitivity and specificity scores. In details, the proposed models achieved an accuracy of 94.44%, surpassing SVM and GCN by 3–8% in AD and HC classification. Additionally, for LMCI and HC classification as well as EMCI and HC classification, the model has accuracies of 93.55 and 92.68%, respectively, outperforming CNN with transfer learning, SVM and GCN by margins ranging from 2 to 10%.

In order to provide readers with a comprehensive understanding of existing publications focused on brain disorder classification using GANs, we have assembled a summary of publications including the publication year, employed imaging modalities, types of datasets and a comparison of classification results between using GAN models for data augmentation and the results where GANs are not used. These details are presented in Table 1. Furthermore, in Table 2, we present the evaluation metrics used to access the quality of data generated by GANs, along with the strengths and limitations of the published models.

**Table 1:** Articles and their notable results in brain disorder classification using GAN.

**Publication**	**Model**	**Imaging modality**	**Dataset/brain disorder**	**Comparison results**
Zhao et al. (2020)	GAN with feature mapping	fMRI/FC	MDD and Schizophrenia	70.1% accuracy, at least 6% higher than other 6 ML model on the classification of MDD patients
				80.7% accuracy, 3–6% higher than other 6 ML models on the classification of schizophrenia patients
Geng et al. (2020)	VAE + GAN	fMRI/FC	ASD, ADHD and ABIDE	For ASD dataset, 87.16% accuracy with GAN compared to 85.35% without
				For ADHD dataset, 87.27% accuracy with GAN compared to 85.06% without
				For ASD-ABIDE dataset, 70.22% accuracy with GAN compared to 67.55% without
Barile et al. (2021)	VAE + GAN	DTI/SC	MS	Achieving 81% F1-score with GAN, compared to 66% F1-score without
Li C. et al. (2021)	BrainNetGAN	DTI/SC	ADNI	Achieving 85.2% accuracy with GAN, compared to 81.9% without
Cao et al. (2023)	BNLoop-GAN	DTI/SC and fMRI/FC	ADNI	Achieving 83.3% with GAN, compared to 81.4% without
Zuo et al. (2023)	HCAL VAE + GAN	DTI/SC	ADNI	Training original data with GAN-generated data, SVM classifier achieved 83.48% accuracy compared to 69.63% without, while BrainnetCNN classifier achieved 84.48% compared to 72.18% without
Pan and Wang (2022)	Cross-modal transformer GAN	DTI/SC and fMRI/FC	ADNI	For the classification between AD and HC, LMCI and HC, EMCI and HC, GAN achieved 94.44, 93.55, and 92.68% accuracy, respectively. There were 3–8%, 3–10%, and 2–10% higher than other deep learning approaches

**Table 2:** Summary of evaluation metrics used for synthetic data and models' strengths and limitations across articles.

**Publication**	**Evaluation metrics**	**Strengths**	**Limitation**
Zhao et al. (2020)	None	New objective loss: feature matching and weight norm regularization for training stability	Generalizability may not be guaranteed across other datasets
		Leave-one-FNC-out method for determining potential biomarkers	No comparison with other deep learning methods
			No evaluation metrics available for synthetic data
Geng et al. (2020)	None	Incorporating VAE for regularization in the latent space and Wasserstein loss to prevent mode collapse and instability during training	No evaluation metrics available for synthetic data
Barile et al. (2021)	Graph metrics	Incorporating VAE for regularization in the latent space and consistency loss to prevent mode collapse and instability during training	Using binary graph, not weight graph
			Significant computation time required for leave-one-out method
			Limited dataset may impact generalizability to other datasets
Li C. et al. (2021)	KL divergenve and MMD	Wasserstein loss with gradient penalty to ensure fast and stable training	Limited dataset may impact generalizability to other datasets
Cao et al. (2023)	None	Wasserstein loss with gradient penalty for stable training	Limited dataset may impact generalizability to other dataset
		Process graph in patch to focus on smaller and more manageable sections	
		Multi-loop algorithm: ranks and selects samples that are easier to learn	No evaluation metrics available for synthetic data
		Effectively integrate mutual information between FC and SC	
Zuo et al. (2023)	MMD	Incorporating VAE for regularization in the latent space and auxiliary classifier loss to prevent mode collapse and instability during training	Limited dataset may impact generalizability to other datasets
		Ensuring both diversity and quality by using cross-connectome aggregating method	
Pan and Wang (2022)	None	Effective fusion scheme: self-attention mechanism to combine both FC and SC information	Limited dataset may impact generalizability to other datasets
		Pair-wise connectivity reconstruction loss for more stable training	No evaluation metrics available for synthetic data

## 5 Limitations of GAN

However, we must not disregard the pertinent concerns associated with the utilization of a GAN model. Firstly, issues such as mode collapse or failure of convergence persist during GAN model training. Therefore, it is crucial to monitor intermediate outputs generated throughout the training process or utilize metrics such as Number of statistically-Different Bins (NDB) approach proposed by Richardson and Weiss (2018) to detect potential mode collapse and in turn promptly address the situation. One of the contributing factors to training instability in GANs is the occurrence of the catastrophic forgetting (CF) problem within the discriminator (Thanh-Tung and Tran, 2020). This problem arises when the parameters learned from previous tasks are destroyed, resulting in disruptions in the training process. Chen et al. (2018) suggested the incorporation of self-supervised tasks as regularization during GAN training. This technique serves to mitigate information forgetting by promoting the learning of meaningful representation. Several regularization techniques, including the weight penalty method (Kurach et al., 2019; Xu et al., 2020), the gradient penalty method (Gulrajani et al., 2017; Hoang et al., 2018), and modified loss function such as WGAN (Arjovsky et al., 2017) or Loss-Sensitive GAN (Qi, 2020) have been proposed to effectively mitigate mode collapse issues in GANs.

Non-convergence and vanishing gradient problems remain as significant challenges in training GAN models. The issues arise when the discriminator performs too well, providing gradient loss near 0 and in turn offering little feedback to the generator for effective learning. Many researchers have taken promising steps to address these challenges. ProgressiveGAN (Karras et al., 2017) has been proposed as a stable approach for training GAN models wherein blocks of layers are added incrementally so that models can learn lower-level features first and later learn ever finer details. The approach has been demonstrated to achieve not only faster converge speed but also to produce higher quality synthetic images compared to GANs without progressive training. Other works propose that GAN be incorporated with other frameworks to increase the stability when training. By leveraging the learned latent space representations of a VAE, GANs can address each other's weaknesses. The incorporation of VAE's latent space information enhances the GAN's capability to generate samples that align with the structured latent space learned by the VAE, achieving more stable and effective training (Geng et al., 2020; Barile et al., 2021).

Optimal hyper-parameters selection is a critical challenge in training GAN models, as different sets of values have been reported to impact the model's performance (Yang and Shami, 2020). For example, varying learning rates can affect the training convergence speed and may lead to divergence, resulting in training instability. Also, a complex architecture might not be suitable for small datasets, while a simpler architecture may not assure optimal performance results for the model. These hyper-parameters include the learning rate, batch size, optimizer for both the generator and discriminator, the number of layers, activation function, loss function and the dropout rate and batch normalization. Grid search and random search are two fundamental hyper-parameter tuning approaches. The former aims to test all combinations of hyper-parameters, while the latter introduces a slight improvement by randomly sampling sets of combinations. On the other hand, Bayesian optimization outperforms those two approaches in terms of computational resources and does not require assumptions about the distribution of the parameters (Yu and Zhu, 2020). One strategy for finding optimal values of hyper-parameters is to use a genetic algorithm (Alarsan and Younes, 2021). Initially, the population is randomly initialized with random parameters, and various steps, such as crossover and mutation, are performed. The process then moves to next population, where only the set of parameters achieving the minimum loss function or maximum accuracy is retained.

When employing GANs for data augmentation in medical imaging, a notable challenge arises: the generated synthetic samples may not faithfully represent real imaging data. Therefore, it is crucial to employ effective evaluation metrics to ensure that the generated samples are beneficial for training ML models. While Barile et al. (2021) employed graph metrics to assess the quality of synthetic graphs, recent studies exhibit a lack of standardization in the evaluation metrics used to gauge the performance of synthetic data generated by GANs for classification.

Furthermore, a significant challenge with deep learning models is their performance variation, while a model may excel with one dataset, it might not generalize as effectively to other datasets. This issue is particularly prominent in the field of medical imaging, where data is sourced from multiple sites featuring scanners from different vendors and varying scan parameters. Strategies for domain adaption have been devised to impart insights from the labeled data domain to analogous but unlabeled domains. The primary aim of these methods is to enhance the performance of models when they are trained across multiple datasets. Medical imaging has seen the integration of GAN models, particularly CycleGAN, to facilitate cross-domain adaption (Rahman et al., 2021; Tomar et al., 2021; Wang et al., 2022). Despite this, the use of this approach to enhance classification performance across multiple datasets, using FC or SC, remains relatively rare. This suggests a novel trajectory that could be pursued in the future.

## 6 Future research

While various progressive solutions have been proposed to mitigate challenges such as mode collapse, non-convergence and instability during the training of GAN models, a trade-off between diversity and quality persists. Balancing between generating diverse outputs and maintaining high-quality results remains a complex task in the ongoing refinement of GAN design and optimization strategies. Future research should delve into more innovative approaches, including new loss objective functions, regularization techniques and hyper-parameters tuning methods to explore the full potential of GANs.

Additionally, the absence of robust and consistent metrics poses imperative challenges in assessing the quality of generated graph brain networks. Future research should prioritize the development of standardized metrics to facilitate the comparison of models, enabling a clear understanding of their performance and ensuring the quality of augmented data generated by GANs, and in turn improving the diagnostic accuracy.

The models listed above primarily test using balanced datasets where the number of patients with disorders is equal to the number of healthy subjects, thereby neglecting the imbalance problem in real-world clinical scenarios. Therefore, future research should place greater emphasis on testing these proposed models on datasets with fewer instances from the patient class, allowing for a better exploration of the potential of GANs. Numerous proposed models continue to rely on CNN models for extracting feature from brain networks, which may not be optimal for capturing the intricate characteristics of graph features. In recent times, the advancement of graph convolutional networks (GCN) within the context of brain networks has showcased their superior performance over conventional CNN designs. Comparative classification performance shows that GCN models excel in learning graph-structured data, surpassing the capabilities of CNN models (Li X. et al., 2021; Deng et al., 2022; Yang et al., 2022). Therefore, the integration of GCN with GANs holds the potential to yield enhanced outcomes.

Furthermore, Transformer (Vaswani et al., 2017), which implements a self-attention mechanism, has recently been proposed as an effective method for learning correlated features in different positions, addressing a limitation where CNN models may fall short. The application of the self-attention mechanism in GANs for data augmentation in the classification of brain disorders using fMRI data has demonstrated improvement (Pan and Wang, 2022). However, the implementation of self-attention in GANs is still in the early stages. Future research can focus on refining its integration into GAN models to improve not only the quality but also the diversity of synthetic data.

## 7 Conclusion

Compared to conventional approaches such as SMOTE or ADASYN, GANs have exhibited a more effective augmentation technique in generating brain networks, leading to a noticeable enhancement in classification performance across multiple articles. The approach has the potential to enhance classification performance, even in situations with imbalanced data distributions (Geng et al., 2020; Barile et al., 2021; Cao et al., 2023). The incorporation of GAN with VAE offers an effective strategy for improving training stability by introducing a posterior distribution. Furthermore, by implementing a fusion scheme between functional and structural networks, as evidenced in prior works by Pan and Wang (2022) and Cao et al. (2023), we can optimize classification performance even further. In addition, the utilization of self-attention mechanisms offers an effective approach to learn key features, contributing to improved model performance (Pan and Wang, 2022).

The methodological primer we provided on GANs, as well as the review of its applications to classification of brain disorders from fMRI data showcases the progress that has happened in the field. However, it also highlights gaps in methodology as well as interpretability that are yet to be addressed. This provides a fertile ground for future work in this area. It would be particularly important for users to understand the range of available methodological choices and the most appropriate workflow for their application while the method developers must be tailor their models to solve real world issues that may be unique to neuroimaging data. Such a synthesis is likely to enrich the field in the future.

## Author contributions

NH: Methodology, Writing – original draft. GD: Methodology, Validation, Writing – review & editing.
